# Primary injection laryngoplasty after chordectomy for small glottic carcinomas

**DOI:** 10.1007/s00405-022-07663-6

**Published:** 2022-10-05

**Authors:** Axelle Felicio-Briegel, Kariem Sharaf, Frank Haubner, Matthias Echternach

**Affiliations:** Department of Otorhinolaryngology, University Hospital, LMU Munich, Marchioninistr. 15, 81377 Munich, Germany

**Keywords:** Injection laryngoplasty, Autologous adipose tissue, Adipose-derived stem cells, Chordectomy, Glottic cancer, Voice outcome

## Abstract

**Objectives:**

The purpose of this study was to analyze the short- and middle-term effects of primary injection laryngoplasty in patients having tumor resection within the same surgery concerning the vocal outcome. Injection laryngoplasty was performed after harvesting autologous adipose tissue via lipoaspiration.

**Methods:**

A prospective study was performed with 16 patients (2 female; 14 male) who received tumor resection and an injection laryngoplasty using autologous adipose tissue during a single stage procedure. Multidimensional voice evaluation including videostroboscopy, patient self-assessment, voice perception, aerodynamics, and acoustic parameters was performed preoperatively, as well as 1.5, 3 and 6 months postoperatively.

**Results:**

Results show an improvement in the roughness–breathiness–hoarseness (RBH) scale, voice dynamics and subjective voice perception 6 months postoperatively. Maintenance of Voice Handycap Index, jitter and shimmer could be observed 6 months postoperatively. There was no deterioration in RBH and subjective voice perception 2 and 6 weeks postoperatively. No complications occurred in the fat harvesting site.

**Conclusions:**

Using the lipoaspiration and centrifugation approach, primary fat injection laryngoplasty shows short-term maintenance und middle-term improvement in voice quality in patients with vocal fold defect immediately after chordectomy 6 months postoperatively. Cancer recurrence rate is comparable to the reported cancer recurrence rate for laryngeal carcinoma and thus not elevated through primary augmentation.

## Introduction

Laryngeal carcinomas are the most frequent head and neck carcinoma [1]. The prognosis for T1 glottic cancers concerning the tumor-related survivals are superior with a 5-year survival rate of 95.5–100% for T1a carcinomas, respectively [2, 3]. For T1a glottic carcinomas, surgery and radiotherapy are equally established as therapy options exhibiting comparable survival rates [4]. Surgical tumor resection using a microlaryngoscopical approach, on one hand, has been shown to be a safe and effective therapy of T1 glottic carcinomas [2, 5]. However, the tumor resection frequently leaves a defect in the vocal folds with patients often having an insufficient glottal closure and thus impairment of vocal function [6]. Furthermore, CO_2_-lasers are frequently used for the surgical removal. Such lasers produce heat resulting in scar tissue formation preventing a sufficient vocal fold oscillation with the consequence that the fragmentation of the transglottic air stream could be inappropriate. On the other hand, radiotherapy frequently reduces vocal fold oscillation with the consequence of voice impairment comparable to surgical tumor resection [7, 8]. When comparing both treatment modalities, tumor resection offers better larynx preservation rate and quicker voice recovery than radiotherapy [9]. Furthermore, during surgery full tumor resection can be controlled by frozen section analysis.

Because of the overall great survival rates concerning glottic T1 laryngeal carcinomas, the non-survival-related factors influencing the post-therapeutically quality of life—such as vocal function—are of great importance. During the last decades, the social necessity to communicate has grown with many employees relying on their voice to accomplish their job [10, 11]. Consequently, for many patients impairment of post-therapeutically vocal function not only results in a diminished quality of life but also in problems concerning the fulfillment of the work-related vocal demands. Therefore, much effort is given to provide early rehabilitation, especially after laryngeal tumor surgery.

Besides, for voice rehabilitation other surgical techniques including laryngoplasties and thyreoplasties [12–14] have been established. For laryngoplasties which augment the vocal folds, many different synthetic or autologous fillers such as polytetrafluoroethylene (Teflon), calcium hydroxylapatite (CaHa), collagen, hyaluronic acid and fat have been used [15–17]. In comparison with other materials, autologous fat has the advantage that no graft rejection is to be expected in comparison with synthetic fillers which can cause local inflammation and foreign body reaction [15]. Laryngoplasties using lipoinjection have been described to be a safe and efficient technique in unilateral vocal fold paralysis, lasting 6 months up to 1 year [16]. Frequently, surgical techniques are performed in a time interval relative to the tumor-related surgery to have a better morphological tumor control. However, due to the time interval the vocal folds frequently become stiff because of scarring which eventually limits the medialization effect for laryngoplasties. Therefore, during the last years, early fat augmentation immediately after removal of the tumor within the same surgical session has been established [18]. It has been assumed that this would result in a better vocal function after the healing process due to the fact that the persisting part of the vocal folds is mostly still soft which permits a greater amount of medialization. However, the vocal outcome after early vocal fold laryngoplasty immediately after tumor resection has not yet been investigated, in detail.

## Materials and methods

After approval from the local ethical committee (project number: 20–563) for a period of 16 months, 16 patients with Tcis, T1 or T2 laryngeal carcinoma were included into a prospective study design. All patients received a surgical tumor resection using a CO_2_ laser at 6 Watt Superpulse (AcuPulse™, Lumenis, Dreieich, Germany) in a microlaryngoscopically approach and general anesthesia by a single surgeon specialized in phonosurgery with a phonosurgical experience of more than 20 years. All patients received at least a chordectomy type III. Removal of the entire tumor was ensured through immediate frozen section analysis during surgery. Table 1 shows the patient characteristics. 10 min before fat harvesting 5 ml of Ultracain 2%^®^ Suprarenin^®^ were injected in the abdominal subcutis. Fat was harvested through lipoaspiration from the abdomen either from the right, left or both periumbilical regions. The fat harvesting set by Spiggle & Theis (Spiggle & Theis Medizintechnik GmbH, Overath, Germany) was used, requiring only a small incision from 1 to 2 mm. With the fat harvesting needle with an inside diameter of 1.2 mm the subcutaneous fat was then rasped and aspirated into a 10 ml syringe. Afterward, the fat was centrifugated for 5 min at 3000 rpm. After centrifugation and removal of the oily and bloody fraction, only the stromal vascular fraction was injected. For fat injection, the stromal vascular fraction was transferred into 1 ml syringes and the fat injection needle with an inside diameter of 0.70 mm at the tip was used. The fat was injected lateral to the thyroarytenoid muscle of the side receiving chordectomy. All patients received solely one sided injection laryngoplasty. The quantity of the injected fat was chosen by the surgeon depending on the visual medialization with an overcorrection of approximately 10–20%.

**Table 1:** TNM, gender, lateralization, chordectomy and oncological follow-up of the study population

T-classification	C_IS_	T1a	T1b	T2
Gender	Female (1)	Female (1)	Female (0)	Female (0)
	Male (1)	Male (11)	Male (1)	Male (1)
Lateralization	Right (1)	Right (7)	Bilateral (1)	Right (1)
	Left (1)	Left (5)		
Chordectomy according to Remacle et al. [24]	Type III (2)	Type III (12)	Type Va (1)	Type Vb (1)
Cancer or C_IS_ recurrence	0	1	0	1
Control laryngoscopy	1	11	1	1

Multidimensional evaluation of the vocal function was performed using the protocol of the European Laryngological Society [19]. The protocol included patient self-assessment [Voice Handicap Index (VHI)] in the German translation [20, 21]; subjective voice perception), voice perception by a phoniatrician (RBH (roughness–breathiness–hoarsness) scale [22]), aerodynamics [maximum phonation time (MPT) and spirometry (ZAN, Oberthulba, Germany)], and acoustic parameters (Goettingen hoarseness diagram including the jitter, shimmer and Glottal-to-Noise-Excitation Ratio for the vowels /a,e,i,o,u,ae/ in 4 conditions (normal, low pitch, high pitch and loud phonation), Wevosys, Forchheim, Germany). Furthermore, for all patients voice range profiles (Wevosys, Forchheim, Germany) with a sound level meter (Voltcraft, Hirschau, Germany) positioned 30 cm from the lips were evaluated. From these measures the Dysphonia Severity Index (DSI) [23] was established. Vocal fold oscillation patters were recorded using transnasal digital videostroboscopy (Xion GmbH, Berlin, Germany). Voice evaluation was performed preoperatively and at 1.5, 3 and 6 months. At 2 weeks postoperatively, solely RBH scale and laryngostroboscopy were performed. At all appointments patients were asked to rate their voice on a four-level scale from normal to severely affected as well as to rate their daily vocal impairment from no impairment to sever impairment with limitations in day-to-day activities.

Statistical analysis and Wilcoxon signed rank test as well as Mann–Whitney *U* test was performed via Excel (Microsoft, Redmond, USA) and R version 4.1.2 (The R Foundation for statistical computing, Vienna, Austria).

## Results

The median age was 65 years (min. 53–max. 87 years) and the median Body Mass Index 23 kg/m^2^ (min. 16–max. 36 kg/m^2^). Regarding the type of chordectomy, one patient received a type Vb chordectomy according to Remacle et al. [24] due to extension to the subglottic area, one patient obtained type Va chordectomy due to extension to the contralateral vocal fold, the other patients all received type III chordectomy. Except from 2 patients, all patients received control laryngoscopy in general anesthesia 4–12 weeks after surgery. The 2 patients not receiving control laryngoscopy were one patient with C_IS_ and one with extremely wide cancer free margins. In two patients there was a postoperative small laryngeal granuloma which was resected during the control laryngoscopy. At the 12 week appointment, 2 patients had to be excluded from further analysis, one due to cancer recurrence in the control laryngoscopy and one due to C_IS_ in the control laryngoscopy.

Regarding safety, one patient was subject to supraglottic swelling due to extremely difficult intraoperative adjustability and, therefore, required intraoperative tracheostomy. This patient was excluded from further analysis, postoperatively. Another patient required surgery for monopolar cauterization due to bleeding during a hypertonic episode a few hours after surgery. No further complications occurred, specifically no complications occurred in the area of the fat harvesting. Preoperatively all patients completed full multidimensional voice evaluation. At 6 weeks postoperatively, full voice evaluation is available for 12 patients and videostroboscopy and RBH scale for 15 patients. At 3 months postoperatively, 12 patients received full voice evaluation and 13 patients stroboscopy and RBH scale. At 6 months postoperatively 8 patients completed full voice testing and 9 patients stroboscopy and RBH scale.

**Fig. 1:**
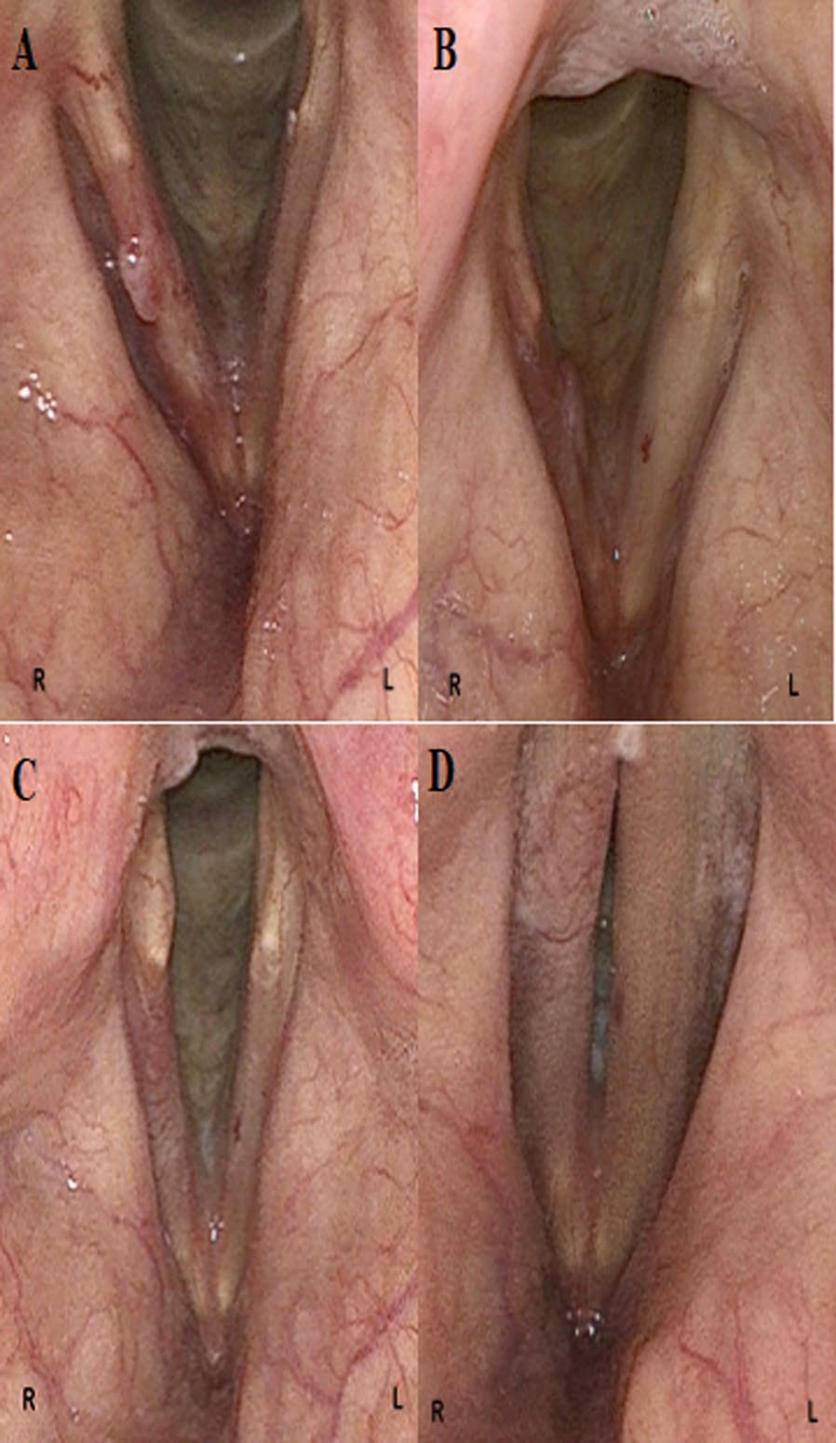
Patient with T1a-carcinoma of the right vocal fold. **A** Preoperative state. **B** 2 weeks postoperatively, a slight hyperemia of the right vocal fold can be seen. **C** 12 weeks postoperatively, scar formation can be seen on the right vocal fold. **D** 26 weeks postoperatively, the right vocal fold shows slightly less volume than the left vocal fold. Scar formation has occurred quite medialized, enabling good glottal closure in videostroboscopy

Figure 1 documents a representative case of a patient with a T1a carcinoma of the right vocal fold. This patient received type III chordectomy with primary injection laryngoplasty. Figure 1 shows the preoperative state of the vocal fold, as well as the state 2, 12, and 26 weeks postoperatively. 26 weeks postoperatively, the right vocal fold shows almost the same volume as the left vocal fold, though scarring has occurred quite medialized, enabling good glottal closure in the videostroboscopy.

### Fat harvesting and injection

**Fig. 2:**
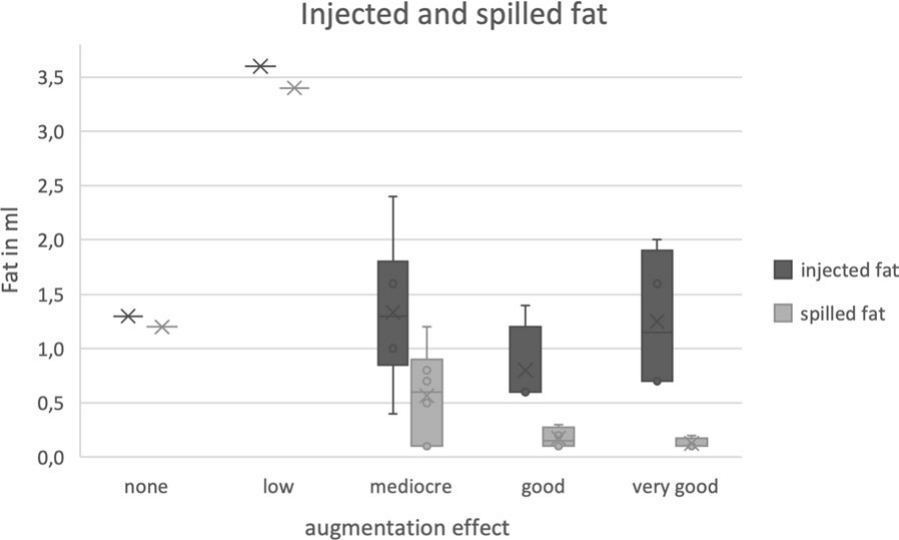
Clustered boxplot of the injected and spilled fat in context with the medialization effect

The median volume of harvested fat was 5.15 ml (min. 4.0 ml; max. 9.0 ml). The median volume of injected fat was 1.15 ml (min. 0.4 ml; max. 3.6 ml) and of the fat spilling out of the vocal fold after injection 0.2 ml (min. 0.1 ml; max. 3.4 ml). Thus, the medium volume, which persisted in the vocal fold was 0.6 ml (min. 0.1 ml; max. 1,9 ml). The medialisation effect was considered low by the surgeon in 1 case, mediocre in 6 cases, good in 4 cases and very good in 4 cases. In one case—the patient requiring tracheostomy—there was no augmentation effect at all, as shown in Fig. 2. No patients needed further surgery for voice improvement during the observation period. No complications occurred in the fat harvesting site.

### Vocal results

**Fig. 3:**
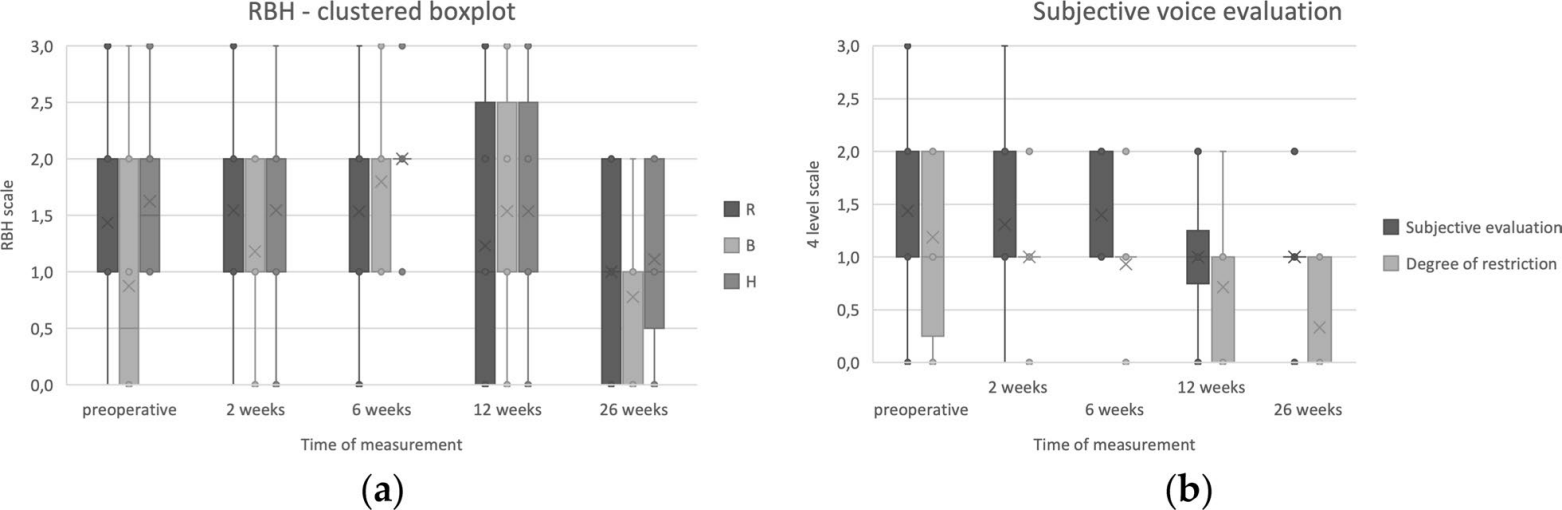
**a** Clustered boxplot of the RBH scale of all patients for the time of measurements preoperative, 2, 6, 12 and 26 weeks. **b** Boxplot for subjective voice evaluation at the time of measurement preoperative, 6, 12 and 26 weeks

In the perceptive RBH scale in Fig. 3 conservation of the preoperative voice state can be found with improvement at 6 months postoperatively. When performing Wilcoxon signed rank test, significant difference was found for preoperative breathiness and 6 weeks (*p* = 0.005; 95% confidence interval (CI) − 2.00 to − 1.00) as well as 12 weeks (p = 0.028; 95% CI – 2.5 to − 0.5). Furthermore, significant difference was found for preoperative hoarseness and 6 weeks (*p* = 0.042; 95% CI – 0.15 to – 0.00005). No significance was found for other measures. The subjective voice perception was rated as equal at 2 and 6 weeks postoperatively and better 12–26 weeks postoperatively, as shown in Fig. 3.

**Fig. 4:**
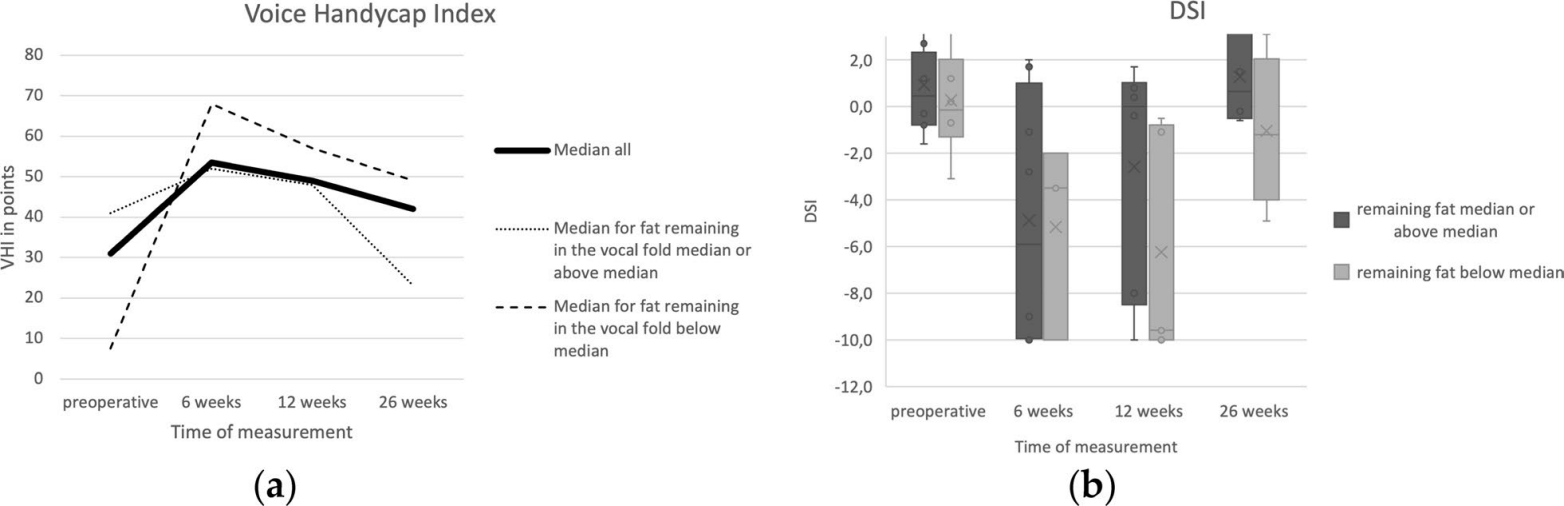
**a** Median VHI for time of measurements preoperative, 6, 12 and 26 weeks. Median VHI for all patients as well as median VHI for the patient group with the remaining fat in the vocal fold being median or above and the patient group with the remaining fat in the vocal fold being below median. **b** Clustered boxplot of DSI in relation to the remaining fat in the vocal fold for the time of measurements preoperative, 6 weeks, 12 weeks and 26 weeks. The median fat remaining in the vocal fold being 0.6 ml

The median VHI was 31 (min. 2; max. 64) preoperatively, 54 (min. 2; max. 75) at 6 weeks postoperatively, 49 (min. 3; max. 63) at 12 weeks, 42 (min. 0; max. 49) at 26 weeks, as shown in Fig. 4. No statistical significance was found. When comparing the patient group having at least median fat (0.6 ml) remaining in the vocal fold intraoperatively and the patient group having below median remaining in the vocal fold, a difference in median VHI can be observed.

When performing Wilcoxon signed rank test there was a significant difference between the preoperative DSI and the DSI at 6 weeks (p = 0.014; 95% CI 0.95–10.14) as well as the DSI at 12 weeks (*p* = 0.019; 95% CI 0.55–8.59). No significant difference was found for 26 weeks. When analyzing the individual values this can be attributed mainly to the MPT, which is decreased at 6 and 12 weeks postoperatively. When dividing the study population in 2 groups in relation to the remaining fat in the vocal fold, better results can be observed for the group with remaining fat volume being median or above median, as seen in Fig. 4. Difference between the groups was not significant.

The vital capacity, MPT, vocal dynamics and fundamental frequency can be found in Table 2. When performing Wilcoxon signed rank test there is a significant difference for vital capacity between the preoperative state and the state at 26 weeks (*p* = 0.034; 95% CI 0.035–1.06). Furthermore, significant difference between the preoperative MPT and the 6 week MPT (*p* value 0.036; 95% CI 0.23–6.76) was found as well as significant difference between the preoperative fundamental frequency and at 6 weeks (*p* = 0.0053; 95% CI − 32,00 to − 700). No significant difference was found for preoperative MPT and 12 week or 26 week MPT or vocal dynamics.

**Table 2:** Median, minimum and maximum for vital capacity, maximum phonation time, vocal dynamics, fundamental frequency and phonation quotient preoperative and 6, 12 and 26 weeks postoperatively

	Preoperative			6 weeks			12 weeks			26 weeks		
	Median	Min	Max	Median	Min	Max	Median	Min	Max	Median	Min	Max
Vital Capacity (l)	3.43	1.84	4.80	3.53	2.36	4.29	3.13	1.42	4.91	2.58	1.89	4.74
Maximum phonation time (sec.)	11.94	4.76	26.35	8.90	3.00	20.55	9.51	3.10	15.15	10.71	5.20	25.45
Vocal dynamics (dB)	21	3	31	14	0	30	18	0	27	26	13	34
Fundamental frequency (Hz)	140	111	200	152	137	211	158	91	244	158	91	219

**Fig. 5:**
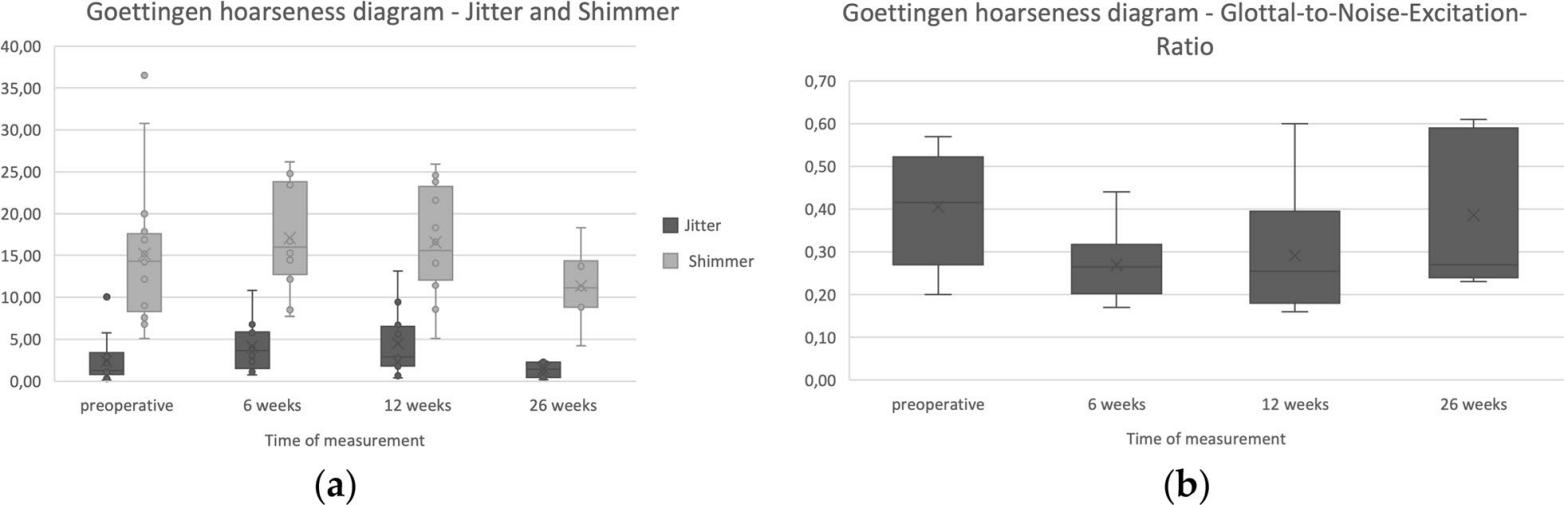
Boxplots for Goettingen hoarseness diagram at the time of measurement preoperative, 6, 12 and 26 weeks. **a** Clustered boxplot for Jitter and Shimmer **b** Boxplot for Glottal-to-Noise Excitation Ratio

The results of the Goettingen hoarseness diagram are displayed in Fig. 5. When performing Wilcoxon signed rank test the difference between the preoperative Glottal-to-Noise-Excitation Ratio and at 6 weeks (*p* = 0.0068; 95% CI 0.035–0.205) as well as 12 weeks (*p* = 0.017; 95% CI 0.10–0.22) was significant. There was no significant difference at 26 weeks as well as Jitter and Shimmer.

### Stroboscopy

Preoperatively 11 patients showed no relevant amplitude nor mucosa wave in the area of the vocal fold mass lesion and 4 patients revealed an impairment of the oscillatory amplitude. 26 weeks after surgery, there were 2 patients with normal, 2 patients with impaired amplitude and 5 patients without any oscillatory amplitude. Complete vocal fold closure could be found for 10 patients preoperatively and also 10 patients at 26 weeks after surgery.

## Discussion

In the present study, the vocal outcome was analyzed after primary laryngoplasty immediately after chordectomy using the stromal vascular fraction of autologous adipose tissue. In general, it has been shown that after a time interval of 6 months, most of the vocal function could be preserved.

Chordectomy types III or higher have been shown to be frequently associated with increased impairment of vocal function [4]. The main alternative to primary laryngoplasty immediately after tumor resection is secondary laryngoplasty or thyreoplasty after a time interval of 6–12 months [13, 25]. One consequence of the secondary approach is that during this time interval many patients suffer from poor voice quality after chordectomy. The presented data exhibit that the primary laryngoplasty after confirmation of cancer free margins in the frozen section preserves most of vocal function with a relative low risk. In this respect, the cancer recurrence rate of 12% seems to be in line with other reports: Peretti et al. report a cancer recurrence rate of 12% and Eckel et al. observed a recurrence rate of 13.9% [4, 26]. In addition, the postoperative granuloma rate of 12% seems to be in accordance with the literature regarding complications after laser surgery of glottic cancer [27]. Furthermore, the observed complications in our study were not related to the liposuction or laryngoplasty.

The use of autologous adipose tissue for injection laryngoplasty has already been proved to be effective in the treatment of insufficient glottal closure, for example, in unilateral vocal fold paralysis [28]. In the presented study, the use of autologous adipose tissue was found to be a safe and effective treatment for primary injection laryngoplasty directly after chordectomy. The fat harvesting procedure showed no complications in the presented study, being a low risk and cost-effective technique for acquisition of autologous material for injection laryngoplasty. If further injection laryngoplasties are needed, fat harvesting can be repeated, representing an almost infinite reservoir of material. In recent studies, the stromal vascular fraction, containing adipose derived stem cells, is said to positively affect wound healing through mechanisms involving migration of fibroblasts and tubulogenesis of endotheliocytes [29]. Its properties to prevent scar formation and promote regeneration of injured vocal fold have been praised [30]. In the presented study, an amelioration or normalization of amplitude could be observed in 4 patients. Whether this has to be attributed to stem cell effects cannot be proven trough this study and needs further studies. Moreover the effects of stromal vascular fraction on cancer growth stimulation and oncologic safety have been discussed with studies showing no increased tumor growth in breast cancer [31, 32]. On the other hand one study analyzing tumor cell proliferation of cancer cell lines of head and neck squamous cell carcinoma (HNSCC) has found that adipose stem cell-derived supernatants promote tumor cell proliferation [33]. Other studies did not find increased proliferation of HNSCC under added adipose derived stem cells [34, 35]. We found no increased rate for cancer recurrence in the presented study. In regards of the potential risk of cancer growth stimulation through adipose derived stem cells, ensuring full tumor resection via frozen section analysis was chosen for this study. Long-term data and higher patient numbers have to be acquired for more definite statements.

To the best of the authors’ knowledge, up to now, primary injection laryngoplasty has rarely been evaluated in clinical studies. Only one study analyzed preliminary results of primary laryngoplasty of 14 patients via modulated injection of 0.5 cc per click and compared those results to patients receiving solely chordectomy without injection laryngoplasty. Postoperative voice evaluation was performed solely 9–16 months postoperatively with VHI, GRBAS scale and multidimensional voice program on the sustained vowel /a/. Furthermore, the fat extraction and injection was performed in the same syringe [36]. Villaret et al. found an improvement of VHI as well as a significant improvement of G, B and A for the patients receiving primary injection laryngoplasty. Objective values of multidimensional voice profile showed no significant difference only an improving trend for patients receiving injection laryngoplasty [36]. In the presented study a more detailed voice analysis was performed according to ELS-protocol at various postoperative timepoints and used a new approach of minimally invasive fat harvesting. Furthermore, in the presented study transfer the stromal vascular fraction to a smaller syringe for injection was chosen. This also allows to avoid the injection of oil and blood and select solely the stromal vascular fraction. The median harvested fat in the presented study was clearly lower with a median of 5 ml comparing 8 cc [36].

In our study at the 6 and 12 week appointment, there was a decrease of voice quality in the MPT, VHI, DSI, Goettingen hoarseness diagram with return to the preoperative state at 6 months postoperatively. When analyzing the vocal results at 6 and 12 weeks, an improvement can already be noted at the 12 week appointment. One possible explanation for the decrease in voice quality at the 6 week appointment could be the two patients developing vocal fold granuloma with resection during the control laryngoscopy between the 6 and 12 week appointment. When looking at the data of those two patients an improvement of objective voice evaluation can be seen for the 12 week measurement, after granuloma resection. Due to the small patient number in our study, data of two patients can already have an important influence on the overall outcome. The decrease in voice including RBH at the 12 week measurement in turn could be attributed to a new wound healing after control laryngoscopy, which occurred between the 6 and 12 week measurement. Due to the design of this study those effects cannot be separated from the effect of the injection laryngoplasty. Our findings can confirm the positive trend of voice for patients after primary injection laryngoplasty already 6 months after surgery accordingly to Villaret et al., who found this trend 9–16 months after surgery [36]. Regarding the minor decrease in vocal function 6 and 12 weeks after surgery a study comparing the decrease in voice quality after primary and secondary injection laryngoplasty is necessary. Yet, it has to be shown, if primary injection laryngoplasty is able to reduce the amount of voice decline in comparison with chordectomy without injection laryngoplasty in the first weeks after surgery.

One challenge of secondary augmentation is scarring of the vocal fold, which subsequently cannot be augmented homogeneously by fluids or gel like substances, such as fat or CaHa. Through primary augmentation this problem can be avoided due to the lack of scarring. In the presented study a more medialized scarring of the vocal fold in augmented patients during stroboscopy has been observed, as seen exemplarily in the presented patient in Fig. 1. Measurements of the volume of the vocal fold are only recently possible by laser triangulation [37], which has not been performed in this study. Whether the scarring of the vocal fold occurs more medially compared to chordectomy without primary injection laryngoplasty can thus not be answered by this study alone and represents a limit of this study. Studies comparing injection laryngoplasty of various materials suggest that voice improvement relies more on the improvement of the glottal closure rather than the chosen material [38, 39]. As such a more medialized scarring could lead to a long-term benefit of the voice. Possibly the material chosen to perform primary injection laryngoplasty is less relevant than thought. Other studies described long-term effects of injection laryngoplasty with autologous adipose tissue with continued improvement 12 months after surgery for unilateral vocal fold paralysis [40, 41]. Studies analyzing the dimensions of the vocal fold after primary injection laryngoplasty directly after chordectomy are needed. Should effects rely more on a more medialized scarring, studies need to analyze whether the material chosen to perform primary injection augmentation is relevant. The use of other fillers for lipoinjection, such as polydimethylsiloxane (Vox Implant ®) or CaHa, would also be favorable in regards of the ongoing debate of the potential influence of autologous fat on tumor growth. For patients with unilateral vocal fold paralysis or vocal fold atrophy CaHa has been proven to be as effective as autologous fat regarding long-term effect [39]. Basing on our results, showing transient voice decline at 1,5 and 3 months postoperatively as well as the possible necessity of granuloma resection at 3 months, when choosing other materials than autologous fat, filler remaining in the vocal fold more than 3 months should be chosen. The use of permanently lasting fillers could reduce the potential necessity of re-injection in the future.

Further limiting factors have to be noted for this study. First, all procedures were performed by solely one surgeon. As such a possible influence of the phonosurgical technique cannot be excluded. Moreover, this surgeon chose an over correction rate of 10–20%. Some surgeons chose over injection of 30% [16]. Whether the amount of overcorrection has an influence has yet to be analyzed. To the best of the authors’ knowledge no study comparing the over correction rate of autologous fat for injection laryngoplasty has yet been performed. Possibly the decrease in voice function at 6 and 12 weeks postoperatively can also be attributed to the smaller over correction chosen in this study. Moreover the median age in our study was 65. Thus, a certain degree of age-related vocal fold atrophy is possible, which could impact the postoperative voice result. In our study solely one sided injection laryngoplasty was performed. Bilateral augmentation directly after chordectomy could further improve the voice in patients showing signs of vocal atrophy. Recent studies have shown low complication rate for awake, bilateral injection laryngoplasty for vocal fold atrophy [42]. Studies regarding the complication rate of bilateral medialization in general anesthesia and in a second step studies regarding bilateral injection laryngoplasty for patients with vocal fold atrophy and chordectomy are needed. Fat was harvested solely abdominally in this study. Other studies chose to also harvest fat glutealy [40]. Whether the fat harvesting site has an influence on the outcome or the amount of adipose stem cells is yet unknown. Moreover several studies have investigated the durability of voice improvement after medialization with fat and the rate of fat absorption over time. Fat graft survival has been thought to be 6–9 months, though recent studies suggest that injection laryngoplasty offers long-term graft survival [40]. As this study chose an investigation period of 6 months postoperatively, duration of fat graft survival and long-term outcomes of vocal function cannot be answered. We would like to point out, that tumor size was solely categorized by TNM-classification. More detailed measurements of tumor size is possible by laser triangulation, which was not performed in this study. Whether exact tumor dimensions have an impact on the vocal outcome of primary injection laryngoplasty is not determinable by this study. In addition, this study includes only a small amount of patients only including one T2- and one T1b-carcinoma. Further studies with higher patient counts are needed to validate our findings especially for patients with T1b- and T2-carcinoma. Ideally a randomized controlled study for comparison between primary and secondary injection laryngoplasty should be conducted.

## Conclusions

Primary injection laryngoplasty immediately after chordectomy is a safe and efficient therapy for small glottic carcinoma. The minimally invasive approach to fat harvesting allows for lipoaspiration with minimal impact.

## Data Availability

The data presented in this study are available on request from the corresponding authors. The data are not publicly available due to restrictions of the institutional IRB statement in concordance to European/German legislation on data restriction.

## References

[1] Bray F, Ferlay J, Soerjomataram I, Siegel RL, Torre LA, Jemal A (2018). Global cancer statistics 2018: GLOBOCAN estimates of incidence and mortality worldwide for 36 cancers in 185 countries. CA Cancer J Clin.

[2] De Seta D, Campo F, D'Aguanno V, Ralli M, Greco A, Russo FY (2021). Transoral laser microsurgery for Tis, T1, and T2 glottic carcinoma: 5-year follow-up. Lasers Med Sci.

[3] Haapaniemi A, Koivunen P, Saarilahti K, Kinnunen I, Laranne J, Aaltonen LM (2016). Laryngeal cancer in Finland: a 5-year follow-up study of 366 patients. Head Neck.

[4] Peretti G, Piazza C, Balzanelli C, Mensi MC, Rossini M, Antonelli AR (2003). Preoperative and postoperative voice in Tis-T1 glottic cancer treated by endoscopic cordectomy: an additional issue for patient counseling. Ann Otol Rhinol Laryngol.

[5] Jones TM, De M, Foran B, Harrington K, Mortimore S (2016). Laryngeal cancer: United Kingdom National Multidisciplinary guidelines. J Laryngol Otol.

[6] Peeters AJ, van Gogh CD, Goor KM, Verdonck-de Leeuw IM, Langendijk JA, Mahieu HF (2004). Health status and voice outcome after treatment for T1a glottic carcinoma. Eur Arch Otorhinolaryngol.

[7] van Loon Y, Sjogren EV, Langeveld TP, Baatenburg de Jong RJ, Schoones JW, van Rossum MA (2012). Functional outcomes after radiotherapy or laser surgery in early glottic carcinoma: a systematic review. Head Neck.

[8] Gandhi S, Gupta S, Rajopadhye G (2018). A comparison of phonatory outcome between trans-oral CO2 Laser cordectomy and radiotherapy in T1 glottic cancer. Eur Arch Otorhinolaryngol.

[9] van Gogh CD, Verdonck-de Leeuw IM, Wedler-Peeters J, Langendijk JA, Mahieu HF (2012). Prospective evaluation of voice outcome during the first two years in male patients treated by radiotherapy or laser surgery for T1a glottic carcinoma. Eur Arch Otorhinolaryngol.

[10] Nusseck M, Spahn C, Echternach M, Immerz A, Richter B (2020). Vocal health, voice self-concept and quality of life in german school teachers. J Voice.

[11] Roy N, Merrill RM, Gray SD, Smith EM (2005). Voice disorders in the general population: prevalence, risk factors, and occupational impact. Laryngoscope.

[12] Fleischer SHM (2016). Durch Augmentation wieder gut bei Stimme: Einseitige Recurrensparese. HNO-Nachrichten.

[13] Gandhi S, Gupta S, Bhowmick N, Pandurangi A, Desai V (2019). Autologous fat augmentation in post type III cordectomy patients. Indian J Otolaryngol Head Neck Surg.

[14] Sano D, Matsushima K, Isono Y, Ikui Y, Kinutani Y, Chiba Y (2020). Long-term treatment outcome of type 1 thyroplasty using novel titanium medialization laryngoplasty implant combined with arytenoid adduction for unilateral vocal cord paralysis: single-arm interventional study at a single institution. Laryngoscope Investig Otolaryngol.

[15] DeFatta RA, Chowdhury FR, Sataloff RT (2012). Complications of injection laryngoplasty using calcium hydroxylapatite. J Voice.

[16] Haddad R, Ismail S, Khalaf MG, Matar N (2021). Lipoinjection for unilateral vocal fold paralysis treatment: a systematic review and meta-analysis. Laryngoscope.

[17] Rosen CA (2000). Phonosurgical vocal fold injection: procedures and materials. Otolaryngol Clin North Am.

[18] Haubner F, Lorenz A, Kummer P, Alvarez JCP (2018). A new atraumatic device for liposuction and injection represents a technical advance for primary fat injection after cordectomy. Laryngorhinootologie.

[19] DeJonckere PH, Crevier-Buchman L, Marie JP, Moerman M, Remacle M, Woisard V (2003). Implementation of the European Laryngological Society (ELS) basic protocol for assessing voice treatment effect. Rev Laryngol Otol Rhinol (Bord).

[20] Verdonck-de Leeuw IM, Kuik DJ, De Bodt M, Guimaraes I, Holmberg EB, Nawka T (2008). Validation of the voice handicap index by assessing equivalence of European translations. Folia Phoniatr Logop.

[21] Nawka T, Wiesmann U, Gonnermann U (2003). Validation of the German version of the Voice Handicap Index. HNO.

[22] Ptok M, Schwemmle C, Iven C, Jessen M, Nawka T (2006). On the auditory evaluation of voice quality. HNO.

[23] Wuyts FL, De Bodt MS, Molenberghs G, Remacle M, Heylen L, Millet B (2000). The dysphonia severity index: an objective measure of vocal quality based on a multiparameter approach. J Speech Lang Hear Res.

[24] Remacle M, Eckel HE, Antonelli A, Brasnu D, Chevalier D, Friedrich G (2000). Endoscopic cordectomy. A proposal for a classification by the Working Committee, European Laryngological Society. Eur Arch Otorhinolaryngol.

[25] Piazza C, BolzoniVillaret A, Redaelli De Zinis LO, Cattaneo A, Cocco D, Peretti G (2007). Phonosurgery after endoscopic cordectomies. II. Delayed medialization techniques for major glottic incompetence after total and extended resections. Eur Arch Otorhinolaryngol.

[26] Eckel HE (2001). Local recurrences following transoral laser surgery for early glottic carcinoma: frequency, management, and outcome. Ann Otol Rhinol Laryngol.

[27] Lee M, Buchanan MA, Riffat F, Palme CE (2016). Complications after CO2 laser surgery for early glottic cancer: an institutional experience. Head Neck.

[28] Cantarella G, Mazzola RF, Domenichini E, Arnone F, Maraschi B (2005). Vocal fold augmentation by autologous fat injection with lipostructure procedure. Otolaryngol Head Neck Surg.

[29] Bi H, Li H, Zhang C, Mao Y, Nie F, Xing Y (2019). Stromal vascular fraction promotes migration of fibroblasts and angiogenesis through regulation of extracellular matrix in the skin wound healing process. Stem Cell Res Ther.

[30] Hiwatashi N, Hirano S, Suzuki R, Kawai Y, Mizuta M, Kishimoto Y (2016). Comparison of ASCs and BMSCs combined with atelocollagen for vocal fold scar regeneration. Laryngoscope.

[31] Lee JS, Eo P, Kim MC, Kim JB, Jin HK, Bae JS (2019). Effects of stromal vascular fraction on breast cancer growth and fat engraftment in NOD/SCID mice. Aesthetic Plast Surg.

[32] Mazur S, Zolocinska A, Siennicka K, Janik-Kosacka K, Chrapusta A, Pojda Z (2018). Safety of adipose-derived cell (stromal vascular fraction - SVF) augmentation for surgical breast reconstruction in cancer patients. Adv Clin Exp Med.

[33] Sharaf K, Eggersmann TK, Haider SP, Schwenk-Zieger S, Zhou J, Gires O (2021). Human adipose-derived stem/stromal cells promote proliferation and migration in head and neck cancer cells. Cancers (Basel)..

[34] Danan D, Lehman CE, Mendez RE, Langford B, Koors PD, Dougherty MI (2018). Effect of adipose-derived stem cells on head and neck squamous cell carcinoma. Otolaryngol Head Neck Surg.

[35] Rowan BG, Lacayo EA, Sheng M, Anbalagan M, Gimble JM, Jones RK (2016). Human adipose tissue-derived stromal/stem cells promote migration and early metastasis of head and neck cancer xenografts. Aesthet Surg J.

[36] BolzoniVillaret A, Piazza C, Redaelli De Zinis LO, Cattaneo A, Cocco D, Peretti G (2007). Phonosurgery after endoscopic cordectomies. I. Primary intracordal autologous fat injection after transmuscular resection: preliminary results. Eur Arch Otorhinolaryngol.

[37] Larsson H, Hertegard S (2008). Vocal fold dimensions in professional opera singers as measured by means of laser triangulation. J Voice.

[38] Cohen JT, Benyamini L (2020). Voice outcome after vocal fold injection augmentation with carboxymethyl cellulose versus calcium hydroxyapatite. J Laryngol Otol.

[39] Zelenik K, Formanek M, Walderova R, Formankova D, Kominek P (2021). Five-year results of vocal fold augmentation using autologous fat or calcium hydroxylapatite. Eur Arch Otorhinolaryngol.

[40] Lahav Y, Malka-Yosef L, Shapira-Galitz Y, Cohen O, Halperin D, Shoffel-Havakuk H (2021). Vocal fold fat augmentation for atrophy, scarring, and unilateral paralysis: long-term functional outcomes. Otolaryngol Head Neck Surg.

[41] Fang TJ, Li HY, Gliklich RE, Chen YH, Wang PC, Chuang HF (2010). Outcomes of fat injection laryngoplasty in unilateral vocal cord paralysis. Arch Otolaryngol Head Neck Surg.

[42] Kelly Z, Patel AK, Klein AM (2021). Evaluating safety of awake, bilateral injection laryngoplasty for bilateral vocal fold atrophy. J Voice.

